# Genomics and physiology of Catenibacillus, human gut bacteria capable of polyphenol C-deglycosylation and flavonoid degradation

**DOI:** 10.1099/mgen.0.001245

**Published:** 2024-05-24

**Authors:** Tobias Goris, Annett Braune

**Affiliations:** 1 Research Group Intestinal Microbiology, German Institute of Human Nutrition Potsdam-Rehbruecke, 14558 Nuthetal, Germany

**Keywords:** *Catenibacillus*, flavonoid, human gut microbiota, *Lachnospiraceae*, polyphenol

## Abstract

The genus *Catenibacillus* (family *Lachnospiraceae*, phylum *Bacillota*) includes only one cultivated species so far, *Catenibacillus scindens,* isolated from human faeces and capable of deglycosylating dietary polyphenols and degrading flavonoid aglycones. Another human intestinal *Catenibacillus* strain not taxonomically resolved at that time was recently genome-sequenced. We analysed the genome of this novel isolate, designated *Catenibacillus decagia*, and showed its ability to deglycosylate *C*-coupled flavone and xanthone glucosides and *O*-coupled flavonoid glycosides. Most of the resulting aglycones were further degraded to the corresponding phenolic acids. Including the recently sequenced genome of *C. scindens* and ten faecal metagenome-assembled genomes assigned to the genus *Catenibacillus*, we performed a comparative genome analysis and searched for genes encoding potential *C*-glycosidases and other polyphenol-converting enzymes. According to genome data and physiological characterization, the core metabolism of *Catenibacillus* strains is based on a fermentative lifestyle with butyrate production and hydrogen evolution. Both *C. scindens* and *C. decagia* encode a flavonoid *O*-glycosidase, a flavone reductase, a flavanone/flavanonol-cleaving reductase and a phloretin hydrolase. Several gene clusters encode enzymes similar to those of the flavonoid *C*-deglycosylation system of *Dorea* strain PUE (DgpBC), while separately located genes encode putative polyphenol-glucoside oxidases (DgpA) required for *C*-deglycosylation. The diversity of *dgpA* and *dgpBC* gene clusters might explain the broad *C*-glycoside substrate spectrum of *C. scindens* and *C. decagia*. The other *Catenibacillus* genomes encode only a few potential flavonoid-converting enzymes. Our results indicate that several *Catenibacillus* species are well-equipped to deglycosylate and degrade dietary plant polyphenols and might inhabit a corresponding, specific niche in the gut.

**Impact Statement**

Herein, we provide a comprehensive overview of the genomic equipment of all currently known members of the intestinal *Lachnospiraceae* genus *Catenibacillus*. Two isolated, but previously genomically not described *Catenibacillus* strains were shown to be among the few human gut bacteria encoding a large variety of proposed enzymes catalytically active on flavonoids. Our studies show that the presence of genes encoding polyphenol-converting enzymes conclusively correlates with the catalysed reactions observed. In particular, *Catenibacillus decagia*, not previously described in the literature, was observed to be capable of extensive conversion of polyphenols, including their metabolically stable *C*-glucosides. This demonstrates how the gut bacterial impact on health-promoting effects of dietary polyphenols may be elucidated based on ever-increasing intestinal (meta)genome data.

## Introduction

The human gut is populated by a huge and diverse array of bacterial species. Some of these bacteria are specialized in the conversion and degradation of secondary plant compounds, such as the relatively recalcitrant polyphenols. For example, *Flavonifractor plautii* (formerly *Clostridium orbiscindens*) and *Eubacterium ramulus,* isolated from healthy human faeces, are known to degrade a range of flavonoids [1,7].

As a major group of polyphenols, flavonoids are widespread in plants to fulfil several functions (e.g. protection against irradiation, predators and harmful micro-organisms) and enter the human gut after consumption of vegetables and fruits. Flavonoids have been attributed a broad range of preventive effects on cardiovascular disorders, obesity, cancer and other chronic diseases [8,9].

Most of the ingested flavonoids bear a sugar moiety (often glucose and/or rhamnose), bound either *O*-glycosidically or, in rare cases, *C*-glycosidically to the flavonoid ring structure. The *O*-glycosidic sugar moieties are readily cleaved off from flavonoids by many different gut bacterial species, especially from the genera *Bifidobacterium* and *Bacteroides* [10,12]. By contrast, *C*-glycosidic bonds are very stable and flavonoid *C*-glycosides such as puerarin, vitexin or orientin, found in a range of medicinal plants, are often not degraded in the human gut [13]. However, in the last two decades, a few bacterial strains have been isolated and characterized to deglycosylate *C*-glucosides of flavonoids and other polyphenols. *Eubacterium cellulosolvens*, mainly present in cattle rumen, but also detected in the human gut, was capable of deglycosylating flavone *C*-glucosides [14]. Two isolates from faeces of healthy adults were able to deglycosylate puerarin: strain PUE, a *Dorea* species [15], and strain CG19-1, subsequently described as *Catenibacillus scindens* and the first species of this genus [16,17]. In addition, several *Enterococcus* strains and one *Lactococcus* strain were described to *C*-deglycosylate flavonoids [18,21]. Compared to *C. scindens*, all other bacteria are more restricted in their *C*-deglycosylation spectrum: while *C. scindens* deglycosylated 6- and 8-coupled flavone *C*-glucosides and the isoflavone 8-*C*-glucoside puerarin, *E. cellulosolvens* deglycosylated only the 6-*C*-glycosylated flavones, isovitexin and homoorientin (or isoorientin) [14,16] and strain PUE deglycosylated only puerarin [15] (for chemical structures refer to Fig. S1, available in the online version of this article).

Isovitexin and homoorientin were deglycosylated by *E. cellulosolvens* enzymes encoded in the *dfgABCDE* gene cluster [22], while strain PUE employed a similar set of enzymes for puerarin deglycosylation encoded by the gene cluster *dgpABC*. The enzymatic reaction mechanism underlying *C*-deglycosylation by strain PUE was uncovered previously [23,25] followed by structural analyses of DgpBC from strain PUE and the homologous DfgAB from *E. cellulosolvens* [26]. The proposed mechanism of *C*-deglycosylation is a two-step process: the oxidoreductase DgpA oxidizes the glucose moiety of puerarin to yield 3″-oxo-puerarin, which is subsequently deglycosylated by DgpBC [25,26]. Similarly, the DgpA homologue from *E. cellulosolvens*, DfgE, oxidizes homoorientin to 3″-oxo-homoorientin and DfgAB cleaves the 3-oxo-glucose moiety from the latter glycoside [26]. The genes encoding the *C*-deglycosylating enzymes in *C. scindens* have not yet been unveiled. The proteins encoded by *dfgCD* of * C. scindens* were induced in the presence of puerarin and show high sequence similarities to the *dfgCD* gene products within the *dfgABCDE* gene cluster of *E. cellulosolvens*. However, *C*-deglycosylating activity was not observed by the two gene products, and instead DfgCD of both bacteria deglycosylated *O*-coupled flavonoid glucosides [22].

Recently, the genome of *C. scindens* DSM 106146 has been sequenced (GenBank accession number GCA_014202115.1) as part of The One Thousand Microbial Genomes Phase 4 Project [27,28]. In addition, the genome of a human gut bacterial isolate and several intestinal metagenome-assembled genomes (MAGs) classified as members of the genus *Catenibacillus* became available from public databases in recent years. The *Catenibacillus* strain had been isolated from faeces of a healthy UK adult [29] and recently named *Catenibacillus decagia* [30] but not further characterized.

In the present study, we performed a comparative analysis of the currently known *Catenibacillus* genomes with a focus on flavonoid metabolism, especially potential flavonoid *C*-deglycosylation gene clusters. In addition, we comprehensively characterized the novel *C. decagia* strain in comparison to *C. scindens*. Here, our major focus was on the ability of *C. decagia* to cleave *C*- or *O*-coupled glycosides of flavonoids and other polyphenols and to further degrade the resulting aglycones.

## Methods

### Screening for *Catenibacillus* genomes, annotation and phylogenetic analysis

The GTDB [31] was searched for the term ‘*Catenibacillus’* on 30 September 2023. In addition, the Genomes Online Database (GOLD, https://gold.jgi.doe.gov), and the taxonomic data of the four human gut microbial genome catalogues UHGG [32], HRGM [33], the Weizmann Institute of Science collection [34] and the early life gut MAGs catalogue [35] were searched for the term ‘*Catenibacillus*’, and corresponding protein sequences were screened with blast-standalone using queries from the housekeeping gene of DNA gyrase (GyrA) with an identity threshold of 0.8. Potential *Catenibacillus* genomes were downloaded as nucleotide fasta files or, in the case of the genome of *C. scindens* DSM 106146, as a GenBank file and uploaded to the RAST server [36] for further analysis and comparative genomics (if possible with their original annotation preserved). The sequence comparison tool of the RAST server was used to compare up to ten genomes. In addition, BlastKOALA [37] annotation was used for physiological comparisons and the PSORTb server [38] for cellular localization prediction, while we manually annotated specific genes of interest using PaperBLAST [39], the dbCAN server for carbohydrate-active enzymes (CAZymes) [40] and a literature screen for flavonoid-modifying enzymes. Whole genome average nucleotide identity was calculated among the genomes with highest completeness among each species using the OrthoANIu tool [41].

### Screening for genes involved in flavonoid conversion

The amino acid sequences of flavonoid-modifying enzymes [11] were screened against the available *Catenibacillus* proteomes via blast searches of the RAST server [36]. An amino acid identity of at least 31 % over the complete sequence was considered as a hit.

### Chemicals

Polyphenols were purchased from Roth, except for the following: homoorientin, orientin, isovitexin, (+)-taxifolin (PhytoLab); hesperetin, mangiferin (Sigma-Aldrich); hesperetin-7-*O*-glucoside, alphitonin (Extrasynthese), puerarin (LKT Laboratories); daidzein (Acros Organics); and hesperidin (TransMIT Chemicals). Norathyriol was available from a previous study [16]. Astilbin and 3-(3,4-dihydroxyphenyl)lactate were obtained from abcr; 3,4-dihydroxyphenylacetic acid and 3-(4-hydroxyphenyl)propionic acid from Fluka; and 3-(3,4-dihydroxyphenyl)propionic acid from Sigma-Aldrich.

### Bacterial strains and cultivation

*C. decagia* (NCBI strain MGYG-HGUT-00127, Biosample SAMEA5849628; Wellcome Sanger Institute) [29] and *C. scindens* DSM 106146 [16,17] were routinely grown under strictly anoxic conditions in 16 ml Hungate tubes containing 10 ml medium with a gas phase of N_2_/CO_2_ (80 : 20, v/v) at 37 °C for 18–20 h. A modified Reinforced Clostridial Medium without agar and starch (RCM_mod_) was applied, which contained (g l^−1^): yeast extract (3.0), meat extract (10), peptone (10), glucose (5.0), NaCl (5.0), sodium acetate (3.0) and cysteine hydrochloride (0.5). Growth was monitored by measuring the optical density at 600 nm (OD_600_) (SmartSpec Plus Spectrophotometer, Bio-Rad). For cultivation on solid media, Columbia agar with 5 % sheep blood (bioMérieux) or Wilkins–Chalgren Anaerobe (WCA) agar (Oxoid) was used. Agar plates were incubated at 37 °C for up to 6 days in an anoxic workstation (MACS Anaerobic Workstation, Don Whitley Scientiﬁc) containing a gas atmosphere of N_2_/CO_2_/H_2_ (80 : 10 : 10, by vol.).

### Phenotypic and biochemical characterization

Gram staining was performed as described previously [42]. Endospores were stained by the Schaeffer–Fulton stain [43]. Catalase, oxidase and tryptophanase activities were tested with ID colour catalase, oxidase reagent and James reagent (all bioMérieux), respectively. Further biochemical characteristics were determined using the Vitek ANI card or the API rapid ID 32 A and API 20 A identification systems (bioMérieux) according to the manufacturer’s instructions with cells grown for 48 h on sheep blood or WCA agar. Glucose concentrations were enzymatically determined (Glucose/Fructose-Kit, Boehringer Mannheim/R-Biopharm) in the supernatant of bacterial cultures grown in RCM_mod_ for 50 h in triplicate after centrifugation (14 000 ***g***, 5 min, 4 °C). Hydrogen was measured in the gas phase of cultures grown in RCM_mod_ for 72 h using a gas chromatograph (3000A Micro GC, Agilent Technologies) with manual injection of sample aliquots (100 µl) as described previously [44]. Calibration was performed by injection of defined amounts of hydrogen.

### Polyphenol conversion tests

For screening of polyphenol conversion by *C. decagia,* 890 µl RCM_mod_ supplemented with 10 µl of 20 mM polyphenol stock solution (in DMSO, sterile ﬁltered) was inoculated with 100 µl of an overnight culture to an initial OD_600_ of ca. 0.1 (ﬁnal polyphenol concentration, 200 µM). Polyphenols and bacteria incubated separately in medium served as controls. Mixtures were prepared in 1.2 ml strips of the Quali 96 Well Tube System (Kisker Biotech) and incubated at 37 °C in the anoxic workstation. Samples (150 µl) were taken at different time points within 7 days of incubation. For comparative studies with *C. decagia* and *C. scindens*, Hungate tubes containing 5 ml RCM_mod_ supplemented with 50 µl of 20 mM polyphenol stock solution were inoculated with 300 µl of an overnight culture to an initial OD_600_ of 0.015 (ﬁnal polyphenol concentration, 190 µM) and incubated at 37 °C. Samples (ca. 300 µl) were withdrawn with a syringe at different time points within 7 days of incubation.

All incubation experiments were performed in duplicate. Sample aliquots (100–150 µl) were lyophilized (Alpha 2–4, Christ) and extracted with the original volume of methanol/water/acetic acid (70 : 29 : 1, by vol.) or, for aliquots from aloin incubations, with methanol/water (70 : 30, v/v). After centrifugation (12 000 ***g***, 5 min), 20 µl of the supernatant was applied to HPLC analysis.

### HPLC analysis

Polyphenols and their degradation products were analysed by reversed-phase (RP)-HPLC in a Dionex UltiMate 3000 system (Thermo Scientific) using a LiChrospher 100 RP-18 column (250×4 mm, 5 µm) equipped with a guard column of the same material (Merck). Column temperature was kept at 37 °C, and autosampler temperature at 15 °C. Injection volume was 20 µl. Aqueous 2 % (v/v) acetic acid (solvent A) and methanol (solvent B) served as the mobile phase in a gradient mode (5 % B for 4 min, from 5 to 95 % B in 20 min, held at 95 % B for 3 min) at a flow rate of 1 ml min^−1^. Calibration curves of standard reference compounds were used for quantification. Detection was at 250, 280, 290 or 350 nm and UV spectra were recorded in the range of 200–400 nm. For control of the HPLC system and data processing, the Chromeleon software version 6.80 (Dionex) was used.

## Results and discussion

### Overview of *Catenibacillus* genomes and the environmental distribution of *Catenibacillus* species

Screening of GTDB [31] for genomes assigned to the genus *Catenibacillus* revealed ten genomes, all of them derived from bacteria in faecal samples from either human donors or chicken. Two of the genomes were derived from isolates, *C. scindens* DSM 106146 and *C. decagia*, and are high-quality draft genomes with 59 and 36 contigs, respectively. The other eight genomes were assembled from metagenomic data (MAGs). Two further MAGs assigned to *Catenibacillus picagaria* and *Catenibacillus tixagia* were found in the data from a recent publication reporting the infant gut genome catalogue [35]. An isolate genome listed in the National Center for Biotechnology Information (NCBI) database as *Catenibacillus* was assembled from faeces of a Hadza hunter/gatherer individual (NCBI WGS accession CAKRHR01 [45]). However, it showed relatively low average amino acid sequence identity (AAI) to any of the other *Catenibacillus* genomes (whole genome AAI<60 %, Table S1). The AAI values of the other genomes to each other were higher than 66 %, which is the value of the majority of the intra-genus AAIs [46]. Therefore, the Hadza-derived genome cannot be classified unambiguously as a *Catenibacillus* genome and was not included in this study. Altogether, 12 *Catenibacillus* genomes included in further analyses (Table 1) showed a completeness of 71–99 %. While one of the MAGs was assigned to *C. scindens* by the GTDB, the other nine MAGs were classified as five distinct species including *Catenibacillus faecigallinarum* and *C. faecavium*, which both have been described initially as *Candidatus* Scybalocola species [47] but were re-classified by the GTDB. The size of genomes was mainly in the range between 3 and 5 Mb except the genome of *C. decagia* with 6.6 Mb. The two isolate genomes were subjected to a prophage search with the online tool PHASTER [48] and the CRISPRCasFinder [49]. An intact prophage was not detected. One CRISPR region and one CAS gene cluster of the class I, type I-C [49,50] with 30 spacers was detected for *C. scindens*. For *C. decagia*, three CRISPR regions (61, 28 and 90 repeats) and three CAS gene clusters (one type I-C and two type III-B, one of the latter presumably incomplete) were detected.

**Table 1.** Overview of *Catenibacillus* genomes (as designated by GTDB) Taxonomy taken from the GTDB. Accession numbers are from NCBI GenBank or, marked with an asterisk, from the original publication [35]. All data taken from the GTDB genome characteristics table or GTDB-Tk tool data for two MAGs from Zeng *et al*. [35], except the sample source data, taken from the NCBI Bioproject and sequence metadata and protein number from RAST annotation where noted.

	*C. scindens* DSM 106146	*C. scindens* CAM219 (MAG)	*C. decagia*	*C. picagaria* (MAG)	*C. picagaria* (MAG)	*C. picagaria* (MAG)
Accession number	JACHFW000000000.1	CAJFOA000000000.1	CABJAM000000000.1	USFV00000000.1	CAJKGK000000000.1	CokerMO_2019_SRR8692231_bin.17*
GTDB species	*C. scindens*	*C. scindens*	sp902363555	sp900553975	sp900553975	sp900553975
Size (bp)	3 872 981	3 069 210	6 637 753	2 508 861	3 086 790	3 048 155
Completeness (%)	98.99	94.46	99.33	71.22	99.33	94.51
Contamination (%)	0.67	2.01	0.67	0.63	0	0.08
Contig number	59	110	36	387	59	138
GC content (%)	46.75	46.89	47.45	43.49	43.39	43.30
Protein number	3481	2720	5608	2446	2659	2911 (RAST)
Source	Human gut (Germany)	Chicken gut	Human gut (UK)	Human gut (Canada)	Human gut (China)	Human infant gut (NZ)
	*C. tixagia* (MAG)	*C. tixagia* (MAG)	*C. faecavium* CHK196-3395 (MAG)	*C. faecavium* Chicken_17_mag_93 (MAG)	*C. faecigallinarum* CHK178-757 (MAG)	*C. faecigallinarum* Chicken_15_mag_111 (MAG)
Accession number	JAGZDD000000000.1	MurphyR_2019_SRR7351834_bin.5*	DVFP00000000.1	CAJFRQ000000000.1	DVIT00000000.1	CAJFGI000000000.1
GTDB species	sp018369015	sp018369015	*C. faecavium*	*C. faecavium*	*C. faecigallinarum*	*C. faecigallinarum*
Size (bp)	4 008 610	4 655 290	2 998 810	3 934 944	3 387 824	3 276 941
Completeness (%)	81.10	94.63	90.60	99.33	99.33	93.67
Contamination (%)	0.67	1.34	0.67	0.67	1.34	0.84
Contig number	244	340	34	64	66	184
GC content (%)	45.27	45.30	45.59	45.10	46.83	46.83
Protein number	3427	4597 (RAST)	2640	3543	3025	2973
Source	Human gut (USA)	Human infant gut (NZ)	Chicken gut	Chicken gut	Chicken gut	Chicken gut

**Fig. 1.**
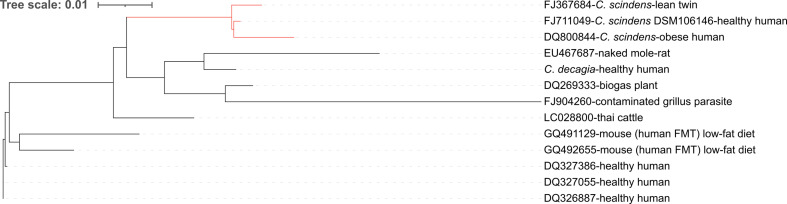
Phylogenetic tree of selected *Catenibacillus* 16S rRNA gene sequences as classified by silva. GenBank accession numbers and sources of sequences are given. For the *C. decagia* 16S rRNA gene sequence, see Data Set S1 (line 2919). Red colour represents *C. scindens* sequences. Taxonomic information at the species level is not available for sequences other than *C. scindens* and *C. decagia*. Minimum sequence length was 750 bp. The corresponding percentage identity matrix is given in Fig. S2. FMT, faecal microbiota transplant. Scale bar, 0.01 substitutions per site.

To unravel the environmental distribution of potential *Catenibacillus* species, we screened the silva 16S rRNA gene sequence database (version SSU_r138.1). As a result, 35 sequences were assigned to the genus *Catenibacillus*, but many of them were too short for reliable classification. None of the MAGs included in Table 1 contained 16S rRNA gene sequences long enough for phylogenetic analysis. We kept the 16S rRNA gene sequences from the silva database with a minimum length of 750 bp and obtained 11 corresponding sequences. Combined with the 16S rRNA gene sequences of *C. scindens* DSM 106146 and *C. decagia*, we constructed an alignment and a phylogenetic tree of 13 sequences (Fig. 1). Most of these sequences were derived from human faecal samples or from related sources, such as faeces from mice colonized with human faecal microbiota. Two of the sequences derived from human samples grouped with *C. scindens*, while the sequence of *C. decagia* did not cluster closely (<97 % sequence identity, see Fig. S2) with another *Catenibacillus* 16S rRNA gene sequence. A few sequences were derived from non-human samples, among them two from animal guts (Thai cattle and naked mole-rat) and one from a biogas plant (Fig. 1). A recent study reported on an increase of *C. scindens* 16S rRNA gene abundance in faeces of ultra-marathon runners after a race [51], but the species designation is questionable since only the V1–V2 region of the 16S rRNA gene was sequenced. Also, the relative abundance of *C. scindens* was very low (mean values <0.1 %) and no data were reported for individual samples of the nine included subjects, hampering meaningful analysis. In three further studies, *Catenibacillus* sequences have been detected in chicken faeces [52,54], but the length of the sequenced 16S rRNA gene region was below 750 bp, impairing meaningful phylogenetic assignment at the species level. A recent study reported elevated levels of *Catenibacillus* abundance in mice fed germinated peanut sprouts, in which the mice showed an alleviation of physical fatigue [55], but the associated sequences were not deposited and, thus, no further analysis could be performed. Taken together, the low numbers of *Catenibacillus* genomes and 16S rRNA gene sequences in the public databases indicate that these are not highly abundant species. Also, recent ultra-deep metagenome sequencing did not reveal *Catenibacillus* sequences in Chinese [56], Mongolian [57] or (see above) Hadza gatherers [45]. The origin of the samples of isolation, metagenome or 16S rRNA gene sequencing suggest that the main habitat of *Catenibacillus* species is the gut of humans and animals, often chicken.

### Genome-guided physiological insight into *Catenibacillus* metabolism

The genomes of the 12 *Catenibacillus* strains (Table 1) were uploaded to the RAST server [36] and BlastKOALA [37] was used for genome-wide comparison and metabolic reconstruction with a focus on the isolate genomes of *C. scindens* and *C. decagia*. The genomes of the two *C. scindens* strains are highly similar, with 2416 of 2923 proteins (RAST annotation) of *C. scindens* CAM219 having at least 97 % amino acid sequence identity to those of *C. scindens* DSM 106146 (Data Set S1), and therefore we refer to both strains of this species if not noted otherwise. The metabolic reconstruction of the *Catenibacillus* genomes indicates a fermentative energy metabolism similar to that of *Clostridium* species. No enzymes involved in anaerobic or aerobic respiration were encoded. Genes for a respiratory complex I, quinone or haem biosynthesis proteins and cytochromes were not detected in any *Catenibacillus* genome. A six-subunit Rnf complex with approximately 50 % amino acid sequence identity to that of *Clostridium ljungdahlii* is conserved in all *Catenibacillus* genomes (reference always given to locus tag numbers of * C. scindens* DSM 106146, HNP82_000177–0182). This membrane-bound protein complex couples the oxidation of ferredoxin to the reduction of NAD^+^ with H^+^- or Na^+^-pumping across the membrane [58]. A putative catalase (HNP82_003074, 50 % amino acid sequence identity to the vegetative catalase of *Bacillus subtilis*) and a small superoxide reductase (122 aa, HNP82_001907; 48 % amino acid sequence identity to the desulfoferredoxin of *Desulfovibrio* species [59]) is encoded in all *Catenibacillus* genomes, except of *C. decagia*, where the catalase is missing. These reactive oxygen species-detoxifying enzymes might facilitate tolerance to low amounts of oxygen, though *C. scindens* DSM 106146 was unable to grow in the presence of atmospheric concentrations of oxygen. A catalase assay was negative for *C. scindens* DSM 106146 [16] and *C. decagia* (see below), which might be due to the strict anoxic conditions applied to grow the tested cultures. A complete glycolysis (Embden–Meyerhof–Parnas) pathway is encoded in all *Catenibacillus* genomes. This contrasts with the inability of *C. scindens* DSM 106146 to degrade glucose [17]. * C. decagia* showed no acid formation from glucose in standard identification tests, but growing cells degraded glucose present in the culture medium (see below). An incomplete tricarboxylic acid (TCA) cycle enzyme equipment, comprising the first four oxidative reactions from oxaloacetate to 2-oxoglutarate, was observed for all *Catenibacillus* genomes. In combination with an encoded Re-citrate synthase (HNP82_002249) [60], this incomplete TCA cycle is similar to that of *Clostridium* spp. In addition, a standard Si-citrate synthase (HNP82_001415) similar to that of *Desulfitobacterium* sp. [61] was identified. The encoded pyruvate–formate lyase (HNP82_001345) for acetyl-CoA and formate generation from pyruvate during anaerobic fermentation shows 65 % amino acid sequence identity to the characterized pyruvate-formate lyase of *Clostridium pasteurianum* [62]. The genomes of *C. scindens* and *C. decagia* contain butyryl-CoA transferase (HNP82_002195–002197) and butyryl-CoA dehydrogenase (HNP82_000334–000338) gene clusters similar to those described for other gut bacteria such as *Eubacterium hallii* and *Faecalibacterium prausnitzii* [63]. This is in line with the observed formation of butyrate by *C. scindens* DSM 106146 [17]. As butyrate-producing bacteria, the two *Catenibacillus* species could contribute to colonic health in their hosts, such as other members of *Lachnospiraceae* [64]. Three gene clusters encoding altogether five cytoplasmic [FeFe] hydrogenases are conserved in the *Catenibacillus* genomes. Three [FeFe] hydrogenases belong to the poorly characterized, putative sensor/regulator type C3 (as classified by the hydrogenase database, HydDB [65]) with a regulatory PAS domain. A second C3-type hydrogenase (HNP82_000320–000321) is encoded upstream of a putative hydrogen-evolving hydrogenase of the B-type (HNP82_000319). Another putative sensing hydrogenase (HNP82_000279–000281) is located upstream of a bi/confurcating A3-type hydrogenase with five subunits (HNP82_000272–000276). These hydrogenases are used to exchange electrons between hydrogen, ferredoxin and dinucleotides, such as NAD(P)^+^. Both *C. scindens* DSM 106146 and *C. decagia* produced hydrogen (between 20 and 40 % of the gas atmosphere after 72 h of cultivation), for which the product of the gene RS01590 or the bifurcating hydrogenase could be responsible. Except that of *C. decagia*, the genomes also encode a [NiFe] hydrogenase of the hydrogen-evolving Ech (energy conserving hydrogenase)-type (HNP82_000649–000654), next to a cluster encoding the [NiFe]-hydrogenase-specific maturases (HNP82_000643–000648).

The gene encoding the core protein involved in sporulation, Spo0A (HNP82_001611), is conserved in all *Catenibacillus* genomes, and the corresponding gene cluster (HNP82_001578–001598) is similar to that of clostridia. For example, Spo0A of *C. scindens* DSM 106146 shows 54 % amino acid sequence identity to Spo0A of *Clostridioides difficile* (AAB05561). Despite the presence of most of the 66 known sporulation genes in *C. scindens* DSM 106146, formation of endospores has not been observed [16]. This might be either due to loss of individual genes or mutations in regulatory elements as observed for *Firmicutes* (now *Bacillota*) inhabiting the human gut [66] or specifically required sporulation conditions. However, *C. decagia* encoding a similar set of sporulation proteins (although the cluster is slightly more spread out including many hypothetical proteins) formed endospores after growth on sheep blood agar for 6 days (see below). Re-testing of *C. scindens* DSM 106146 under identical conditions did not lead to spore formation again. Whether this is due to a different regulation of sporulation or an incomplete set of sporulation proteins in *C. scindens* remains to be evaluated.

All *Catenibacillus* genomes encode enzymes for vitamin B12 (cobalamin) biosynthesis (main cluster proteins, WP_183776114 to WP_183776145), similar to those of *Clostridium* species and to the enzymes involved in the well-described anaerobic biosynthesis by *Salmonella enterica* [67]. Several cobalamin-dependent enzymes are encoded in the *C. scindens* genomes, notably a methionine synthase (MetH, HNP82_001373), and two further methyltransferases (HNP82_000421, HNP_000658), distantly related to characterized bacterial methyltransferases (<30 % amino acid sequence identity). One cobalamin riboswitch could be identified, located upstream of the cobalamin ABC transporter genes *btuCDF* (HNP82_001097–001099).

In general, *C. scindens* and *C. decagia* appear to be genetically well equipped to use different kind of carbohydrates. Enzymes to degrade di- and monosaccharides (sucrose, mannose, fructose, xylose) and possibly starch and/or glycogen are encoded in their genomes. A thorough analysis on the CAZyme gene content in the *Catenibacillus* genomes is presented in the next section. Only a few phosphotransferase systems (PTS) are encoded in *C. scindens*, among those systems with a high confidence annotation for transport of sucrose, trehalose and fructose. Of these, only the sucrose-specific PTS (HNP82_000312) with 42 % amino acid sequence identity to that of *Streptococcus thermophilus* [68] is conserved in all *Catenibacillus* genomes except that of * C. faecigallinarum*. The corresponding *ptsABC* genes are embedded in a sucrose utilization cluster including a sucrose 6-phosphate hydrolase (HNP82_000311) and a fructokinase (HNP82_000310) and are presumably under the control of a sucrose operon repressor, ScrR (HNP82_000313). A putative trehalose-transporting PTS similar to that of *Clostridium perfringens* [69] is encoded next to a trehalose hydrolase and a corresponding repressor (HNP82_000352–000354). This gene cluster and a putative glucose-specific PTS (HNP82_001370), similar to that of *Staphylococcus carnosus* [70], are only conserved in *C. tixagia*, *C. faecavium* and * C. faecigallinarum*. In contrast, a putative fructose-related gene cluster (HNP82_001185–001187, 48 % amino acid sequence identity to the fructose-specific PTS of *Bacillus subtilis*) is only conserved in *C. decagia* and *C. faecavium*. Besides PTS proteins, a potential fucose transporter of the major facilitator superfamily (MFS) (HNP82_001683) is encoded in the *C. scindens* genome. The gene is located next to a glucosidase gene (HNP82_001684) encoding a protein very similar (66 % amino acid sequence identity) to a maltose-hydrolysing enzyme from *Blautia obeum* [71]. In addition, several uncharacterized ABC transporters of the aldouronate transporter family are encoded, but they have low similarity to characterized transporters. The high abundance of genes encoding putative carbohydrate-utilizing enzymes is in contrast to physiological and biochemical investigations, in which *C. scindens* DSM 106146 has not been observed to ferment glucose or other carbohydrates [17]. However, the presence of glucose in the medium enhanced growth [17], suggesting its assimilation into cell material. In contrast to *C. scindens*, *C. decagia* largely degraded glucose contained in the growth medium, although tests were negative for glucose oxidation in standard identification assays (see below). The latter may be due to missing induction of expression of involved genes under growth conditions used in those tests.

Based on genomic analysis, *Catenibacillus* members appear not to be dedicated peptide/amino acid or fatty acid degraders. The genomes do not encode enzymes involved in the Stickland reaction often employed to gain energy from amino acid fermentation, and only a few proteases, peptidases or amino-acid-degrading enzymes are encoded (e.g. a glutamate dehydrogenase, HNP82_002820). Testing for peptide cleavage or amino acid fermentation using bacterial identification systems had revealed solely negative results for *C. scindens* DSM 106146 [17], which was also observed for *C. decagia*, with one exception only (leucine arylamidase, see below). In *C. scindens*, as well as in *C. faecavium* and *C. faecigallinarum*, a urease gene cluster is conserved (HNP82_003036–003043). It resembles the gene cluster of certain *Helicobacter* species, also encoding the rarely included pH-gated urea channel UreI required for gastric *Helicobacter pylori* colonization [72]. However, as a specific periplasmic loop present in UreI from *H. pylori* is missing in the UreI proteins encoded in *Catenibacillus* genomes, these potential gene products more closely resemble the UreI from enteric *Helicobacter* species, such as *Helicobacter hepaticus* [72]. The urease tests were negative for * C. scindens* DSM 106146 [17] and *C. decagia* (see below), but specific growth conditions such as a more acidic growth medium or Ni supplementation were not tested.

### CAZyme analysis based on the genomes of *Catenibacillus* isolates

A CAZyme analysis for potential glycosyl hydrolases and transferases suggests that xylosidases and β-glucosidases play important roles in the metabolism of *C. scindens*. Overall, genes coding for 146 CAZymes were detected in the genome of *C. scindens* DSM 106146 by screening the CAZy database using the dbCAN server (Data Set S1). Of these genes, 49 were predicted by all three algorithmic modules (HMMer, eCAMI and Diamond) employed by the dbCAN server. The highest number of genes coded for members of the CAZy families GT4 (22 genes, nine of which were detected by at least two CAZy modules) and GH39 (21 genes, 14 of which were detected by at least two CAZy modules). Members of the GT4 family are mainly involved in the synthesis of membrane-associated carbohydrates, e.g*.* peptidoglycans, and are often encoded in peptidoglycan synthesis gene clusters. GH39 family members, including β-glucosidases and xylosidases, are rarely encoded in such high numbers in single bacterial genomes as in those of the two *Catenibacillus* isolates. A similarly high abundance has been found only in genomes of uncharacterized bacteria of the *Opitutacea* and *Victivallales* lineages (as per the CAZY database, December 2022). In contrast to the outcome of analysis of its gene equipment, *C. scindens* DSM 106146 tested negative for activity of a range of glycosyl hydrolases including β-glucosidase and xylosidase in previous studies [17].

The CAZyme equipment of *C. decagia* is higher compared to that of *C. scindens*, with 330 detected CAZymes including 89 of high certainty. The higher number might at least partly be explained by its larger genome size. The highest abundance was observed for gene products classified as members of CAZy families GH3 (28 genes, 27 of which were detected by at least two CAZy modules), GH39 (28 genes, 15 of which were detected by at least two CAZy modules), GH109 (27 genes, detected by the HMMer module only) and GH2 (18 genes, detected by at least two CAZy modules). The number of GH3 enzymes encoded in the *C. decagia* genome, mostly β-glucosidases, is amongst the highest number of these CAZymes in single bacterial genomes and comparable to the number of GH3 genes in *Bacteroides cellulosolyticus*. In *Bacteroides* species, many of these CAZymes are secreted and, therefore, supposed to act on extracellular substrates too large to be imported. In contrast, *Catenibacillus* GH3 sequences were not observed to contain signal peptides indicating their intracellular location. Since *C. decagia* tested positive for α- and β-glucosidase, α- and β-galactosidase, and α-arabinosidase activities (see below), several of these GH3 enzymes might be responsible for cleaving off sugar moieties from aromatic compounds, probably including flavonoids or other polyphenols (see next section). The also highly abundant GH78 enzymes encoded in the *C. decagia* genome (16 genes, ten of which were detected by at least two CAZy modules) include potential flavonoid rhamnosidases (see next section). Overall, only four putative extracellular (exo-) CAZymes were detected for *C. scindens* and five for *C. decagia*, mostly distantly related to known enzymes. The *Catenibacillus* exo-CAZymes are probably involved in peptidoglycan/murein synthesis or degradation since their genes are part of corresponding gene clusters. Not classified as exoenzyme by the dbCAN server (which uses the SignalP algorithm for location prediction), but identified by a genome-wide screening via the PSORTb-server, a putative secreted amylase (neopullulanase, HNP82_003152, GH13 family) is encoded in all *Catenibacillus* genomes including MAGs. This enzyme is possibly acting on starch, glycogen or oligosaccharides derived thereof. A similar extracellular amylase of *Thermoactinomyces vulgaris* (TVA1) (32 % amino acid sequence identity) endo-hydrolyses pullulan (a starch-derived oligosaccharide) to yield panose and also acts on cyclodextrans [73]. Only 12 further CAZymes predicted by at least two CAZy modules are conserved (>60 % amino acid sequence identity) among all *Catenibacillus* genomes including two of the GT2 family and three of the GH13 family. Six of the conserved CAZymes are predicted to participate in biosynthesis of the peptidoglycan cell wall. A putative xylosidase (HNP82_001909) is conserved as well, indicating that xylose or similar carbohydrates might serve as growth substrate for *Catenibacillus* strains. Overall, the highly species-specific CAZyme equipment of the *Catenibacillus* members points toward a differing, possibly species’ niche-specific carbohydrate substrate spectrum of this genus. It is known that the gut bacterial CAZyme content may differ due to the dietary intake of polysaccharides by the host and that species-level and even strain-level differences may occur [74].

### Flavonoid conversion potential of *Catenibacillus* strains

**Fig. 2.**
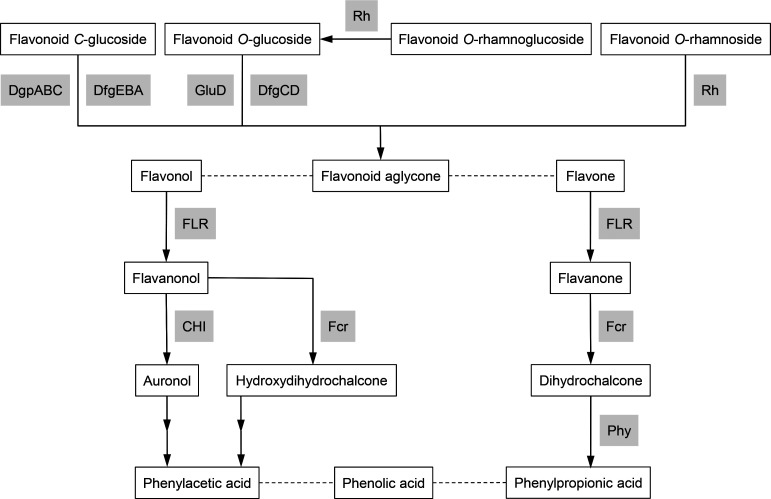
Overview depicting pathways of flavonoid conversion and involved enzymes in human gut bacteria. CHI, chalcone isomerase; DgpABC and DfgEBA, *C*-glucosidases; Fcr, flavanone/flavanonol-cleaving reductase; FLR, flavone reductase; GluD and DfgCD, *O*-glucosidases; Phy, phloretin hydrolase; Rh, rhamnosidase.

We performed a blast-based analysis among all *Catenibacillus* genomes using sequence information of characterized flavonoid-converting enzymes [11] to detect gene products with a potentially similar function. An overview of pathways of flavonoid conversion and involved enzymes in human gut bacteria is provided in Fig. 2. All of the *Catenibacillus* genomes encode potential flavonoid-transforming enzymes (Table 2). The MAGs of *C. faecigallinarum* encode the fewest number of potential flavonoid-converting enzymes, with only two proteins similar to the *C*-glucoside 3″-oxidases DgpA and DfgE plus a potential flavone reductase (FLR). *C. faecavium* MAGs encode, in addition, three different *C*-glucosidases (DgpBC), though their genes are not located adjacent to *dgpA*, in contrast to *dgpABC* being part of a single operon in strain PUE [24]. The occurrence of several DgpBCs suggests a broad *C*-deglycosylation potential of *C. faecavium. C. tixagia* MAGs also encode three DgpBCs and, in addition, several putative ring-cleaving flavanone/flavanonol reductases (Fcr). In contrast, *C. picagaria* does not encode a DgpBC, but several rhamnosidases similar to characterized flavonoid rhamnosidases from human faecal metagenomes [75] (Table 2), suggesting a physiological niche of degrading flavonoid rhamnosides. *C. picagaria* MAGs also encode putative enzymes involved in reductive flavone and flavanone degradation: an FLR, an Fcr and a chalcone isomerase (CHI), but no phloretin hydrolase (Phy). Since the MAGs are incomplete, several genes might have been missed in this analysis. However, this should not be a major issue, because at least two MAGs for each species were available (not differing in the observed flavonoid-modifying enzyme equipment) and at least one of them is close to complete (Table 1).

**Table 2.** Putative flavonoid-converting enzymes encoded in *Catenibacillus* genomes All genomes of each species were combined, since only minor differences between strains were observed. A threshold of 33 % amino acid sequence identity across an alignment coverage of at least 80 % was used. Close orthologues (bidirectional hits with at least 80 % amino acid sequence identity) between *C. scindens* and *C. decagia* are shaded green. Percentage sequence identity to the query sequence (first column with accession numbers) is given in parentheses. Locus tag numbers for *C. scindens* are in parentheses (HNP82_00XXXX), and numbers for *C. decagia* are RAST locus tags (see Data Set S1). Corresponding enzymatic conversions are depicted in Fig. 2.

Bp*Bifidobacterium pseudocatenulatum*Cl*Clostridium ljungdahlii*Cs*C. scindens*Dsp*Dorea* sp. PUEEc*Eubacterium cellulosolvens*Er*Eubacterium ramulus*Fp*Flavonifractor plautii*La*Lactobacillus acidophilus*Lp*Lactobacillus plantarum*MGmetagenome-derived

The MAG of *C. scindens* CAM219 also encodes a flavonoid *O*-glucosidase DfgCD as has been previously characterized in the polyphenol *C*- and *O*-deglycosylating *C. scindens* DSM 106146. In addition, a DfgE gene was detected in *C. scindens* genomes (54 % amino acid sequence identity to DfgE from *E. cellulosolvens* [22]). Also, several potential monomeric flavonoid *O*-glucosidases similar to characterized ones of *Bifidobacterium* species were detected. Several *dgpA* and *dgpBC* gene clusters were also observed. In accordance with the observed degradation of flavone aglycones by *C. scindens* DSM 106146 [16], a putative FLR and two putative Fcrs are encoded in its genome. The high number of potential *C*-deglucosylating enzymes might explain the broad range of *C*-glucoside substrates used. Of the three encoded putative DgpA proteins, one (gene sequence: HNP82_000642) has a very high amino acid sequence identity of 66 % to the characterized DgpA of strain PUE oxidizing puerarin to 3″-oxo-puerarin [26], while the other two (gene sequences: HNP82_002713, HNP82_001555) show intermediate amino acid sequence identity of 39 and 42 %, respectively, to DgpA of strain PUE. The potential strain PUE-specific DgpA encoded by *C. scindens* (HNP82_000642) is a very close orthologue with more than 90 % sequence identity to DgpAs encoded in the MAGs of * C. faecavium* and *C. tixagia*. As in these MAGs, the *dgpA* genes are not adjacent to *dgpBC* in *C. scindens*, in contrast to the cluster arrangement in strain PUE. Four *C*-glucosidases, DgpBC, are encoded by *C. scindens*. One of these putative enzymes (gene sequence: HNP82_001250/1249) is similar to DgpBC of strain PUE (amino acid sequence identity of 67 and 53 %, respectively). These putative enzymes, DgpA and DgpBC, could be involved in puerarin deglycosylation observed for *C. scindens* DSM 106146 [16]. Interestingly, *C. scindens* gene products of *dgpBC* (HNP82_001250/001249) do not have close orthologues in *C. faecavium* or *C. tixagia* (no bidirectional hits for DgpB and only 50 % amino acid sequence identity for DgpC). This variety in the proteins involved in polyphenol *C*-deglucosylation could hint towards a more intricate *C*-deglucosylation physiology, in which different enzyme sets are responsible for the *C*-deglucosylation of different substrates. Both *C. faecavium* and *C. tixagia* might be unable to deglycosylate puerarin, despite encoding an enzyme highly similar to DgpA of strain PUE. This hypothesis may be tested when isolates of these species become available.

According to the potential polyphenol-converting enzymes encoded in the MAGs from chicken gut, *Catenibacillus* species (*C. scindens, C. faecavium, C. faecigallinarum*; Table 1) could play a role in metabolism and, thereby, bioavailability of these compounds when given as feed additives to poultry. Polyphenols including flavonoids are being applied to farm animals as alternative growth promoters replacing antibiotics [76,77]. In chicken, the beneficial effects of fulvic acid on growth performance and immune function through the modulation of gut microbiota, including enrichment of *Catenibacillus*, was recently reported [52].

*C. decagia* encodes by far the most potential flavonoid-converting enzymes including a large number of rhamnosidases, flavonoid-cleaving reductases and *C*-deglycosylating enzyme complexes (Table 2). *C. decagia* encodes DfgAB, DfgCD and DfgE homologues. An exceptional number of 11 genes encoding DgpA homologues were detected in the *C. decagia* genome, one of which is closely related to DgpA from strain PUE (75 % amino acid sequence identity) and a close orthologue (91 % amino acid sequence identity) to DgpA of *C. scindens* (HNP82_000642). Six *dgpBC* gene clusters were found (Table 2). DgpBC_5907/5908_ from *C. decagia* is most similar to DgpBC from strain PUE (54 and 63 % amino acid sequence identity) and no close orthologue to DgpBC from * C. scindens* (HNP82_001250/001249), with only about 60 % amino acid sequence identity.

### Characterization of *C. decagia*

*C. decagia* was isolated from a healthy UK adult [29] and recently named automatically [30], but has not yet been phenotypically characterized. We observed growth of *C. decagia* under strict anoxic conditions only. By cultivation in RCM_mod_ at 37 °C, a maximal OD_600_ of 3 was reached after 24 h (Fig. S3). Cells were non-motile, Gram-positive, ovoid to short rods, 1.8–2.6 µm long and 0.9 µm wide and occurring singly, in pairs or in short chains (Fig. S4a). Colonies formed after 6 days at 37 °C on Columbia sheep blood agar were approximately 1.5 mm in diameter, non-haemolytic, matt grey with a white centre, circular and raised with an entire margin. Cells showed no catalase, oxidase, urease or tryptophanase activity. In standard identification tests, *C. decagia* tested positive for α- and β-glucosidase, α- and β-galactosidase, α-arabinosidase, alkaline phosphatase (only with 2-naphthyl-phosphate using cells grown on sheep blood agar) and leucine arylamidase, but did not degrade carbohydrates or amino acids (Tables S2–S4). However, growing cells of *C. decagia* degraded glucose (28 mM) present in RCM_mod_ nearly completely within 48 h (Fig. S3). Centrally located elliptical and bulging endospores were observed after growth on sheep blood agar for 6 days (Fig. S4b).

### Conversion of polyphenols by *C. decagia* and *C. scindens*

The ability of *C. decagia* to convert polyphenolic plant compounds was elucidated by incubating *C*-glucosides of flavonoids and other polyphenols, flavonoid *O*-glycosides and flavonoid aglycones with growing cells of this bacterium (Table 3, chemical structures in Fig. S1). The flavone *C*-glucosides, vitexin, isovitextin and homoorientin, were completely degraded to the respective hydroxyphenylpropionic acids within 6 h of incubation, while the corresponding degradation of orientin was somewhat slower (Table 3). The individual phenolic acids are specifically formed from the B- and C-rings of the flavonoid basic structure, whereas phloroglucinol results from the A-ring. However, the latter is difficult to detect by RP-HPLC analysis and probably further degraded to short-chain fatty acids [1]. The isoflavone *C*-glucoside puerarin was not converted within 7 days of incubation. Regarding other polyphenolic *C*-glucosides, *C. decagia* was found to deglycosylate the xanthone mangiferin to norathyriol within 6 h, but did not convert the anthracenone derivative aloin within 7 days of incubation (Table 3).

**Table 3.** Polyphenol depletion and metabolite formation within 6 and 24 h by *Catenibacillus decagia* Values are means of duplicate incubations. 3,4-DPA, 3-(3,4-dihydroxyphenyl)acetic acid; 3,4-DPP, 3-(3,4-dihydroxyphenyl)propionic acid; 4-HPP, 3-(4-hydroxyphenyl)propionic acid; H7G, hesperetin-7-*O*-glucoside; na, not applicable.

Polyphenol	Class	% Polyphenol depletion		Final product [Intermediate(s)]
		within 6 h	within 24 h	
***C*-Glucosides**				
Vitexin	Flavone	100	na	4-HPP
Isovitexin	Flavone	100	na	4-HPP
Orientin	Flavone	81	100	3,4-DPP
Homoorientin	Flavone	100	na	3,4-DPP
Puerarin	Isoflavone	1.5	0	na
Mangiferin	Xanthone	100	na	Norathyriol
Aloin	Anthracenone derivative	0	0	na
***O*-Glycosides**				
Apigenin-7-*O*-glucoside	Flavone	100	na	4-HPP
Luteolin-7-*O*-glucoside	Flavone	100	na	3,4-DPP
Daidzin	Isoflavone	62	100	Daidzein
Phloridzin	Dihydrochalcone	22	96	4-HPP
Rutin	Flavonol	89	100	3,4-DPA [Quercetin]
Naringin	Flavanone	100	na	4-HPP
Hesperidin	Flavanone	46	100	3,4-DPP[H7G, hesperetin]
Astilbin	Flavanonol	100	na	3,4-DPP
**Aglycones**				
Quercetin	Flavonol	100	na	3,4-DPA
Taxifolin	Flavanonol	100	na	3,4-DPA
Alphitonin	Auronol	100	na	3,4-DPA
Hesperetin	Flavanone	16	100	3,4-DPP

All of the tested flavonoid *O*-glycosides and aglycones were completely degraded within 6–48 h of incubation to the corresponding hydroxyphenolic acids, except for daidzin, which was only deglycosylated to daidzein (Table 3). Thus, *C. decagia* not only catalysed *O*-deglycosylation but also cleavage of the C-ring of flavonoids belonging to flavones, flavanonols, flavonols and flavanonols. The flavonoid *O*-glycosides included glucosides, rhamnoglucosides (α1→2 linked in naringin or α1→6 linked in rutin and hesperidin) and a rhamnoside (astilbin). Based on the obtained data, a clear correlation of conversion rates with the type or position of attached sugar moieties or the flavonoid class could not be found. However, the methoxylated flavonoids, hesperidin and its aglycone hesperetin, were degraded most slowly, but likewise the dihydrochalcone glucoside phloridzin. Beside the actual affinity and activity of involved enzymes, the efficiency of degradation may also depend on solubility and uptake of individual polyphenols.

**Fig. 3.**
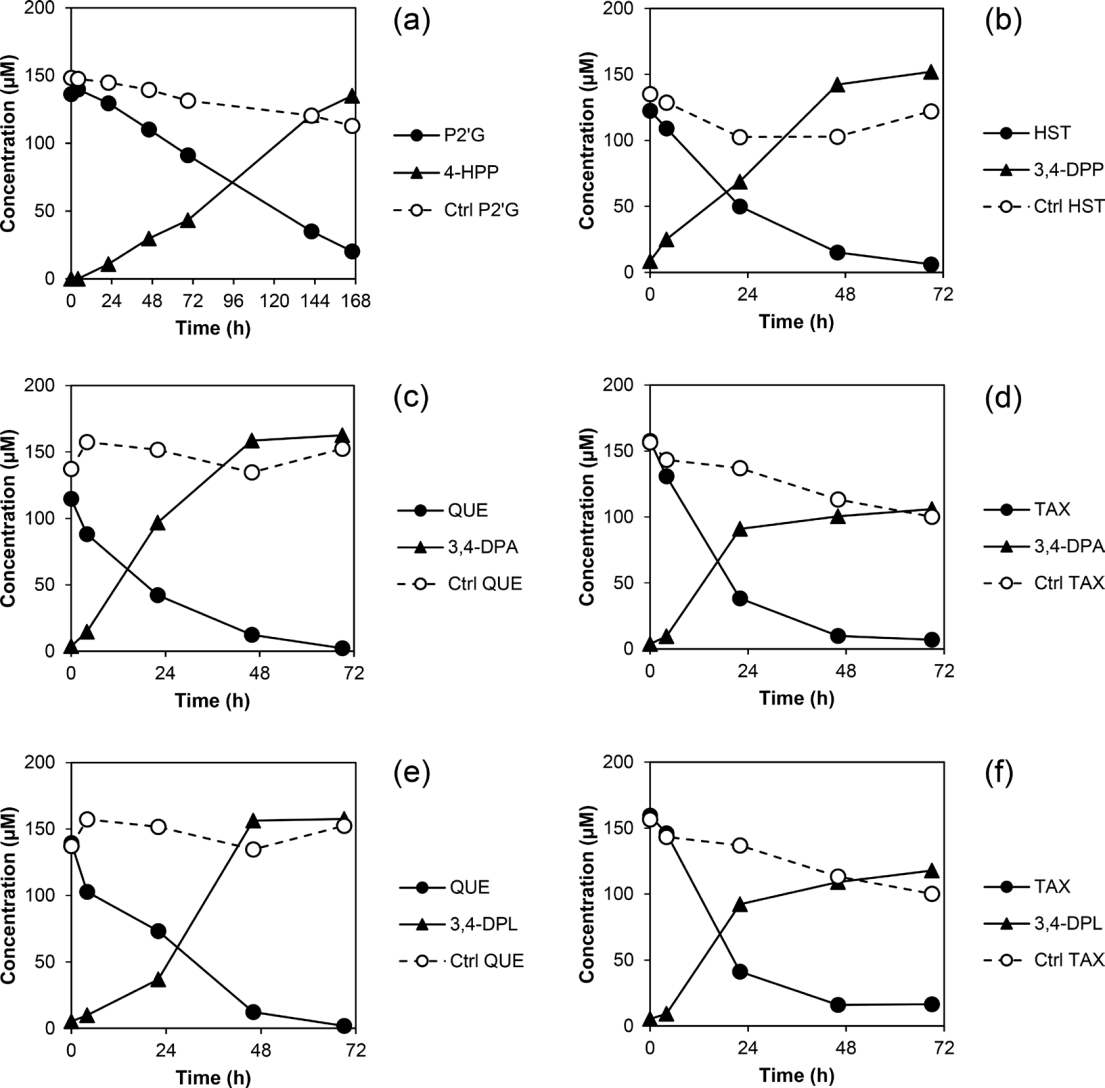
Conversion of selected flavonoids by the two *Catenibacillus* isolates. *C. scindens* converted (**a**) phloridzin (P2’G) to 3-(4-hydroxyphenyl)propionic acid (4-HPP) and (**b**) hesperetin (HST) to 3-(3,4-dihydroxyphenyl)propionic acid (3,4-DPP). *C. decagia* converted both (**c**) quercetin (QUE) and (**d**) taxifolin (TAX) to 3,4-dihydroxyphenylacetic acid (3,4-DPA), whereas *C. scindens* converted (**e**) quercetin (QUE) and (**f**) taxifolin (TAX) to 3-(3,4-dihydroxyphenyl)lactic acid (3,4-DPL). The symbols represent the means of duplicate incubations. As controls (Ctrl), flavonoids were incubated in medium.

The results obtained in conversion tests with polyphenolic *C*-glucosides for *C. decagia* were identical to those reported for * C. scindens* DSM 106146 [16], except of its inability to deglycosylate puerarin. The flavonoid *O*-glucosides, apigenin-7-*O*-glucoside, luteolin-7-*O*-glucoside and daidzin, are known to be degraded also by *C. scindens* [16], while no data were available for the remaining flavonoids listed in Table 3. Therefore, we tested the conversion of selected flavonoid *O*-glycosides and aglycones in parallel by *C. decagia* and *C. scindens* at larger scale. Phloridzin was degraded by *C. scindens* to 3-(4-hydroxyphenyl)propionic acid (4-HPP) (Fig. 3a) as observed for *C. decagia* (Table 3), but at a lower rate (incomplete conversion within 7 days). Hesperetin was degraded by *C. scindens* to 3-(3,4-dihydroxyphenyl)propionic acid (3,4-DPP) (Fig. 3b) as observed for *C. decagia* (Table 3). In contrast to *C. decagia,* hesperidin and astilbin were not converted by *C. scindens* within the maximal incubation period of 7 days, which may be explained by the absence of rhamnosidases in this bacterium. Genes encoding potential rhamnosidases are widespread in the *C. decagia* genome, but were not detected in the genome of *C. scindens* DSM 106146 as discussed above (Table 2). Other conversion reactions (C-ring cleavage, demethylation) appear not to be possible without initial deglycosylation of the flavonoids. Quercetin and its reduced metabolite, taxifolin, were degraded by both *C. scindens* and *C. decagia* at similar rates, but with formation of different final products (Fig. 3c–f). *C. decagia* converted quercetin, taxifolin and also alphitonin to 3,4-dihydroxyphenylacetic acid (3,4-DPA) (Table 3, Fig. 3c, d). This indicates degradation of quercetin via taxifolin and alphitonin by the same pathway as has been described for *E. ramulus* [1,3]. The conversion of both quercetin and taxifolin by *C. scindens* did not yield any 3,4-DPA, but another product, which was subsequently identified as 3-(3,4-dihydroxyphenyl)lactic acid (3,4-DPL) by comparison with an authentic standard compound (Fig. 3e, f). So far, phenyllactic acids have not been described as products of bacterial flavonoid degradation. For metabolism of tyrosine and phenylalanine by anaerobic gut bacteria, an oxidative pathway leading to (4-hydroxy) phenylacetic acid and a reductive pathway leading to (4-hydroxy) phenyllactic acid have been described, both starting from (4-hydroxy) phenylpyruvic acid [78]. Similarly, the observed formation of either 3,4-DPA or 3,4-DPL could have started from the hypothetical intermediate 3,4-dihydroxyphenylpyruvic acid, which may result from alphitonin cleavage beside phloroglucinol.

Overall, *C. decagia* is capable of converting a wide range of flavonoids by *C*- and *O*-deglycosylation, demethylation, reduction and C-ring cleavage. Hence, this novel *Catenibacillus* species is similarly active as *C. scindens*, and, moreover, is able to metabolize flavonoid rhamnosides. With respect to *C*-deglycosylation, *C. decagia* also cleaved the xanthone mangiferin, but not the isoflavone puerarin. Puerarin with its glucose moiety attached to the C-8 position is also not deglycosylated by *E. cellulosolvens* or the *Enterococcus* strains found to be capable of cleaving C-6 and/or even C-8-coupled flavone-*C*-glucosides [14,19,21]. This indicates that the differing basic structure of the isoflavone aglycone prevents its use as a substrate.

The gene equipment of *C. scindens* and *C. decagia* as evaluated by analysing their genomes (Table 2) is in agreement with their polyphenol-converting enzyme activities observed herein or in previous studies. The only exception is the absence of a gene in *C. scindens* that encodes a CHI similar to that of *E. ramulus* and involved in flavonol/flavanonol conversion in the latter bacterium [79]. According to our data, *C. scindens* degrades flavonols/flavanonols by the identical initial steps as *E. ramulus* [1]. However, enzyme sequences could differ, in particular for distantly related species. Although this may also limit the prediction of flavonoid-directed activities for the other *Catenibacillus* strains based on our MAG analysis (Table 2), these bacteria appear to be less active in flavonoid conversion. Hence, *C. scindens* and *C. decagia* could preferentially impact host health by increasing the bioavailability of dietary polyphenols and their bioactive metabolites.

### Conclusions

Gut bacteria play a crucial role in the metabolization of dietary flavonoids and other polyphenols, thereby contributing to the proposed health effects of these plant compounds in humans and also farm animals. Knowledge on the bacteria involved is still limited even though their enzymes may activate or inactivate ingested polyphenols by formation of a range of metabolites. Our study provides detailed experimental data on polyphenol conversion by two members of the genus *Catenibacillus*, the novel * C. decagia* and the previously isolated *C. scindens* DSM 106146. The two strains belong to the few characterized human gut bacteria that are able to cleave polyphenolic *C*-glucosides and degrade flavonoid aglycones. Based on the bacterial ﬂavonoid-converting enzymes known to date, large-scale screening of human gut metagenomes enables identification of other relevant bacterial species responsible for flavonoid conversion [11,80]. *C. scindens* DSM 106146 was identified and characterized following classical activity-driven isolation [16,17]. Herein, we analysed in detail the genomes of this first described *Catenibacillus* strain and the second isolate, *C. decagia*, with a focus on genes encoding potential flavonoid-converting enzymes. While the related gene equipment matched overall well with the experimentally determined flavonoid-converting capabilities of the two *Catenibacillus* isolates, a large number of potentially polyphenol-active enzymes encoded in *C. decagia* point towards an even broader substrate spectrum, probably attributed to the large structural variety of polyphenols and their corresponding glycosides. In addition, we present a first glimpse into an understudied bacterial genus probably belonging to low-abundance gut microbiota via the analysis of MAGs of other *Catenibacillus* members. Overall, the results of our study demonstrate that genome analysis may provide significant information on gut bacteria involved in dietary flavonoid conversion. However, to further improve their reliable identification, the database of experimentally characterized enzymes and pathways needs to be further extended.

## supplementary material

10.1099/mgen.0.001245 Uncited Supplementary Material 1.

10.1099/mgen.0.001245 Uncited Table S1.

## Abbreviations

AAI: average amino acid sequence identity
CAZyme: carbohydrate-active enzyme
CHI: chalcone isomerase
3,4-DPA: 3,4-dihydroxyphenylacetic acid
3,4-DPL: 3-(3,4-dihydroxyphenyl)lactic acid
3,4-DPP: 3-(3,4-dihydroxyphenyl)propionic acid
Fcr: flavanone/flavanonol-cleaving reductase
FLR: flavone reductase
GTDB: Genome Taxonomy Database
4-HPP: 3-(4-hydroxyphenyl)propionic acid
MAG: metagenome-assembled genome
OD_600_: optical density at 600 nm
Phy: phloretin hydrolase
RCM_mod_: modified reinforced clostridial medium
