# Supplementary material for: Genomics and physiology of Catenibacillus, human gut bacteria capable of polyphenol C-deglycosylation and flavonoid degradation
Source: Microb Genom. 2024 May 24;10(5):001245. doi: 10.1099/mgen.0.001245 (PMC11170127; doi:10.1099/mgen.0.001245)
Supplement: Uncited Supplementary Material 1. [file mgen-10-01245-s001.pdf]

Genomics and physiology of *Catenibacillus*, human gut bacteria capable of polyphenol *C*-deglycosylation and flavonoid degradation

Tobias Goris and Annett Braune

SUPPLEMENTARY MATERIAL

Supplementary Table S1. Whole genome average amino acid identities (AAI) of selected *Catenibacillus* species genomes and the Hadza-derived genome. The most complete genome of each species (GTDB taxonomy) was subjected to the RAST sequence-based comparison and the resulting table-file to the Newman AAI calculator.

	C. scindens	*C. faecavium*	*C. faeci-gallinarum*	*C. decagia*	*C*. sp. 900553975	*C*. sp. 018369015
C. faecavium	75.0	---				
C. faecigallinarum	75.0	85.4	---			
C. decagia	67.1	66.4	67.8	---		
C. sp. 900553975	66.1	67.3	67.4	67.6	---	
C. sp. 018369015	72.7	72.0	72.9	69.0	68.1	---
Hadza MAG	59.6	59.5	59.9	59.4	59.5	59.4

Supplementary Table S2. Results of biochemical testing of *C. decagia* using the Vitek ANI card. Bacteria for inoculation were grown on sheep-blood agar. -, negative reaction; w, weak reaction; +, positive reaction

**Substrate**	**Result**
4-Nitrophenyl phosphate	-
4-Nitrophenyl phosphate choline	-
4-Nitrophenyl-β,D-galactopyranoside	w
4-Nitrophenyl-α,D-galactopyranoside	+
4-Nitrophenyl-β,D-glucopyranoside	+
4-Nitrophenyl-α,D-glucopyranoside	+
4-Nitrophenyl-β,D-glucuronide	-
4-Nitrophenyl-β,D-lactoside	-
4-Nitrophenyl-α,D-mannopyranoside	-
4-Nitrophenyl-α,L-fucopyranoside	-
4-Nitrophenyl-β,D-fucopyranoside	-
4-Nitrophenyl-β,D-xylopyranoside	-
4-Nitrophenyl-α,L-arabinofuranoside	-
4-Nitrophenyl-*N*-acetyl-glucosaminide	-
N-Benzoyl-DL-arginine 4-nitroanilide	-
L-Leucine 4-nitroanilide	-
L-Proline 4-nitroanilide	-
L-Alanine 4-nitroanilide	-
L-Lysine 4-nitroanilide	-
γ-Glutamyl 4-nitroanilide	-
Triphenyl tetrazolium	-
Arginine	-
Urea	-
Glucose	-
Trehalose	-
Arabinose	-
Raffinose	-
Xylose	-

Supplementary Table S3. Results of biochemical testing of *C. decagia* using the API Rapid ID 32 A identification system. Bacteria for inoculation were grown on Columbia sheep-blood agar (COS) or Wilkins-Chalgren Anaerobe agar (WCA). -, negative reaction; +, positive reaction

**Substrate**	**Result**	
	**COS**	**WCA**
Urea	-	-
L-Arginine	-	-
4-Nitrophenyl-α,D-galactopyranoside	+	+
4-Nitrophenyl-β,D-galactopyranoside	+	+
4-Nitrophenyl-β,D-galactopyranoside-6-phosphate-2CHA	-	-
4-Nitrophenyl-α,D-glucopyranoside	+	+
4-Nitrophenyl-β,D-glucopyranoside	+	+
4-Nitrophenyl-α,L-arabinofuropyranoside	+	+
4-Nitrophenyl-β,D-glucuronide	-	-
4-Nitrophenyl-*N*-acetyl-β,D-glucosaminide	-	-
D-Mannose	-	-
D-Raffinose	-	-
L-Glutamic acid	-	-
4-Nitrophenyl-α,L-fucopyranoside	-	-
Potassium nitrate	-	-
L-Tryptophan	-	-
2-Naphthyl-phosphate	+	-
L-Arginine-β-naphthylamide	-	-
L-Proline-β-naphthylamide	-	-
L-Leucyl-L-glycine-β-naphthylamide	-	-
L-Phenylalanine-β-naphthylamide	-	-
L-Leucine-β-naphthylamide	+	+
Pyroglutamic acid β-naphthylamide	-	-
L-Tyrosine-β-naphthylamide	-	-
L-Alanine-β-naphthylamide	-	-
L-Glycine-β-naphthylamide	-	-
L-Histidine-β-naphthylamide	-	-
L-Glutamyl-L-glutamic acid-β-naphthylamide	-	-
L-Serine-β-naphthylamide	-	-

Supplementary Table S4. Results of biochemical testing of *C. decagia* using the API 20 A identification system. Bacteria for inoculation were grown on Columbia sheep-blood agar (COS) or Wilkins-Chalgren Anaerobe agar (WCA). -, negative reaction

**Substrate**	**Result**	
	**COS**	**WCA**
L-Tryptophane	-	-
Urea	-	-
D-Glucose	-	-
D-Mannitol	-	-
D-Lactose (bovine origin)	-	-
D-Saccharose	-	-
D-Maltose	-	-
Salicin	-	-
D-Xylose	-	-
L-Arabinose	-	-
Gelatin (bovine origin)	-	-
Esculin ferric citrate	-	-
Glycerol	-	-
D-Dellobiose	-	-
D-Mannose	-	-
D-Melezitose	-	-
D-Raffinose	-	-
D-Sorbitol	-	-
L-Rhamnose	-	-
D-Trehalose	-	-

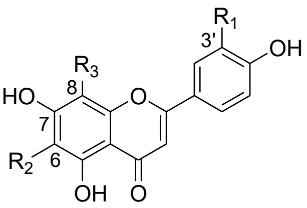

Homoorientin: R_1 = OH, R_2 = glucosyl, R_3 = H
Orientin: R_1 = OH, R_2 = H, R_3 = glucosyl
Luteolin: R_1 = OH, R_2, R_3 = H
Isovitexin: R_1 = H, R_2 = glucosyl, R_3 = H
Vitexin: R_1, R_2 = H, R_3 = glucosyl
Apigenin: R_1, R_2, R_3 = H

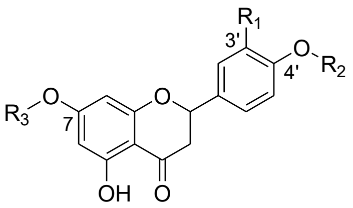

Naringin: R_1, R_2 = H, R_3 = neohesperidosyl
Hesperidin: R_1 = OH, R_2 = CH_3, R_3 = rutinosyl
Hesperetin: R_1 = OH, R_2 = CH_3, R_3 = H

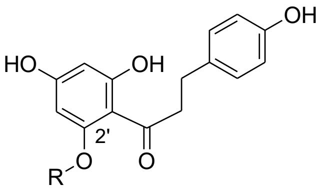

Phloridzin: R = glucosyl
Phloretin: R = H

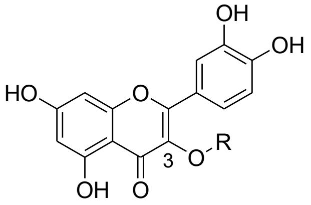

Rutin: R = rutinosyl
Quercetin: R = H

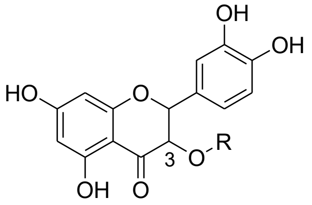

Astilbin: R = rhamnosyl
Taxifolin: R = H

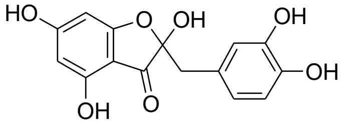

Alphitonin

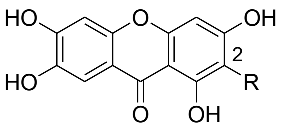

Mangiferin: R = glucosyl
Norathyriol: R = H

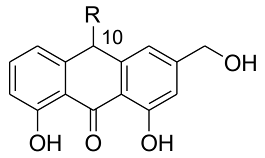

Aloin: R = glucosyl

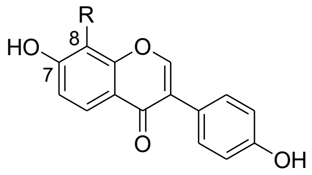

Puerarin: R = glucosyl
Daidzein: R = H

Supplementary Fig. S1. Structures of flavonoids and other polyphenols used in the study.

	% Identity												
	1	2	3	4	5	6	7	8	9	10	11	12	13
1: FJ904260.1 (1465 bp)	100.00	88.45	88.37	89.53	89.53	89.60	90.97	94.65	90.50	90.72	89.43	89.87	90.83
2: GQ491129.1 (1360 bp)	88.45	100.00	96.76	97.42	97.42	97.49	94.22	93.05	91.04	93.82	92.42	93.16	93.16
3: GQ492655.1 (1380 bp)	88.37	96.76	100.00	98.74	98.74	98.67	95.25	94.89	91.75	94.56	93.47	94.48	94.33
4: DQ327055.1 (1354 bp)	89.53	97.42	98.74	100.00	100.00	99.93	96.50	96.83	92.68	95.64	94.60	95.20	95.57
5: DQ326887.1 (1354 bp)	89.53	97.42	98.74	100.00	100.00	99.93	96.50	96.83	92.68	95.64	94.60	95.20	95.57
6: DQ327386.1 (1354 bp)	89.60	97.49	98.67	99.93	99.93	100.00	96.43	96.96	92.76	95.71	94.68	95.27	95.64
7: LC028800.1 (1514 bp)	90.97	94.22	95.25	96.50	96.50	96.43	100.00	98.04	93.00	96.50	94.79	95.40	95.98
8: DQ269333.1 (767 bp)	94.65	93.05	94.89	96.83	96.83	96.96	98.04	100.00	96.31	96.61	95.30	95.41	95.95
9: EU467687.1 (1272 bp)	90.50	91.04	91.75	92.68	92.68	92.76	93.00	96.31	100.00	96.15	92.30	93.00	93.24
10: *C. decagia* (1538 bp)	90.72	93.82	94.56	95.64	95.64	95.71	96.50	96.61	96.15	100.00	94.90	95.51	96.22
11: DQ800844.1 (1391 bp)	89.43	92.42	93.47	94.60	94.60	94.68	94.79	95.30	92.30	94.90	100.00	98.31	98.76
12: FJ367684.1 (1359 bp)	89.87	93.16	94.48	95.20	95.20	95.27	95.40	95.41	93.00	95.51	98.31	100.00	99.26
13: FJ711049.1 (1454 bp)	90.83	93.16	94.33	95.57	95.57	95.64	95.98	95.95	93.24	96.22	98.76	99.26	100.00

Supplementary Fig. S2. Percent identity matrix based on *Catenibacillus* 16S rRNA sequence comparisons. For details on strain and sequence sources refer to the corresponding phylogenetic tree in Fig. 1.

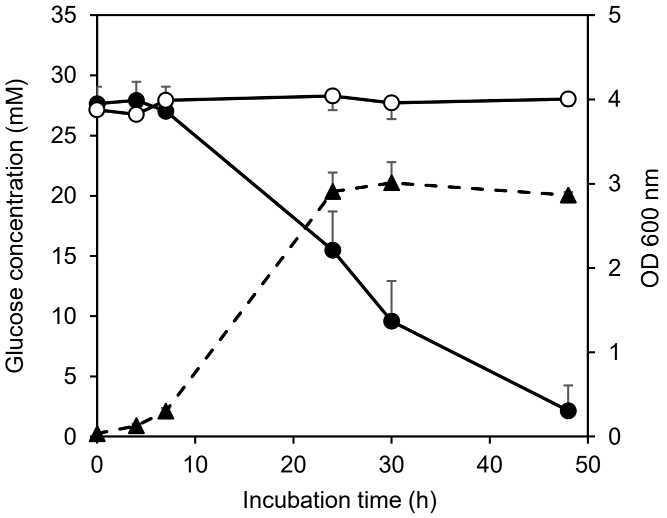

Supplementary Fig. S3. Time course of glucose fermentation by growing cells of *C. decagia* (●). Bacterial growth is indicated by a broken line (▲) referring to the *y* axis on the right. Control incubations were performed with medium in the absence of bacteria (○). The symbols represent the means of triplicate experiments. Error bars indicate SD.

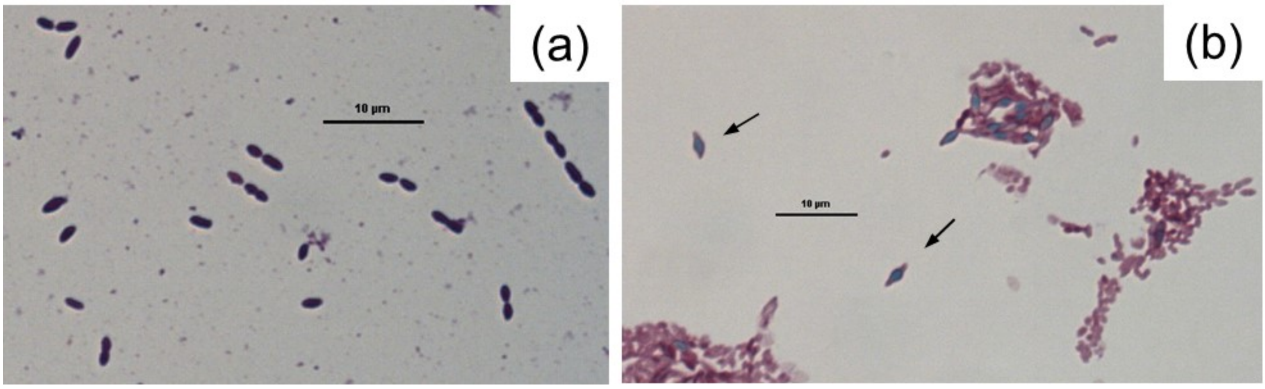

Supplementary Fig. S4. Light micrographs of *C. decagia*. (a) Gram staining of cells cultured in RCM_{mod} medium. (b) Endospore staining of cells grown on sheep-blood agar. Arrows depict single endospore-containing cells. Bar, 10 µm.
